# Preference for diagnosing and treating renal colic during pregnancy: a survey among Chinese urologists

**DOI:** 10.1038/s41598-024-53608-w

**Published:** 2024-02-05

**Authors:** Shidong Deng, Dayong Guo, Lingzhi Liu, Yurou Wang, Kuilin Fei, Huihui Zhang

**Affiliations:** 1 Department of Urology, The First Affiliated Hospital, Hengyang Medical School, University of South China, Hengyang, 421001 Hunan China; 2 Department of Obstetrics, Xiangya Hospital, Central South University, Changsha, 410008 Hunan China; 3 The First School of Clinical Medicine, Southern Medical University, Guangzhou, 510515 Guangdong China; 4 Institute of Hospital Administration, University of South China, Hengyang, China

**Keywords:** Renal colic, Pregnancy, Diagnosis, Treatment, Survey, Urology, Urogenital diseases, Urinary tract obstruction

## Abstract

To explore the preference for diagnosing and treating renal colic during pregnancy among Chinese urologists. A questionnaire was designed using the Sojump^®^ platform. WeChat, the largest social networking platform in China, was used to distribute the questionnaire to urologists at hospitals of all levels in China. In total, 110 responses were included. Of the respondents, 100.0% used ultrasound to diagnose renal colic during pregnancy, followed by magnetic resonance imaging (17.3%) and low-dose CT (3.6%). Phloroglucinol (80.9%) and progesterone (72.7%) were the most commonly used antispasmodics and analgesics. Opioid analgesics were not commonly used (12.7%). Most of the respondents (63.6%) indicated that no more than 20% of the patients needed surgical intervention. If surgery was unavoidable, 95.5% preferred temporary renal drainage, including ureteral stenting (92.7%) and percutaneous nephrostomy (2.7%). However, some respondents still preferred definitive stone treatment, such as ureteroscopy lithotripsy (3.6%) and percutaneous nephrolithotomy (0.9%). Moreover, there were no differences in the choices of urologists with different professional titles regarding diagnostic tools, most therapeutic medications, or surgical methods (*p* > 0.05). Ultrasound is the preferred tool for diagnosing renal colic during pregnancy. Low-dose CT is still not widely accepted. Pregnant patients with renal colic are initially treated conservatively. Urologists prefer ureteral stenting when there are clinical indications for intervention.

## Introduction

Renal colic, a urological emergency, is common during pregnancy and caused by a series of nonobstetric factors, including urinary calculus^1^. Renal colic affects 1:200 to 1:2000 of individuals^2^. For both female parents and foetuses, renal colic can lead to serious harmful effects^3^. In addition, renal colic may be confused with abdominal pain of obstetric origin^4^. Many physiological and anatomical changes that affect disease diagnosis and treatment occur in the female body during pregnancy^5^. Therefore, the management of renal colic during pregnancy is challenging for urologists.

Ultrasonography (US) is the primary tool for diagnosing pregnant patients suspected of having renal colic^6^, and magnetic resonance imaging (MRI) is used as a second-line procedure. Due to the teratogenic effects of high X-ray emission, computed tomography (CT) should be avoided in pregnant women. However, a previous study showed that radiation doses less than 50 mGy are not associated with the risk of malformation or miscarriage during pregnancy, indicating that low-dose CT is also an option for diagnosis^7^.

Although conservative treatment of renal colic during pregnancy is the first choice, 25–30% of patients eventually require intervention due to intractable symptoms or other indications^8^. Either renal drainage or definitive stone treatment is performed^2^. Renal drainage is advantageous in that it is simple and relatively less risky, but it provides a temporary solution and patients may require frequent stent replacement^9^. In definitive stone treatment, the stone is completely removed; however, the procedure is riskier and more costly^10^. Both intervention types are feasible, but the preferred type of intervention is the subject of debate and depends on the personal preference and comprehensive assessment of the doctor.

The management of pregnant women with renal colic depends on the doctor involved. In the present study, we aimed to explore the preference of Chinese urologists for diagnosing and treating renal colic during pregnancy.

## Patients and methods

Using the Sojump^®^ platform, we designed a 17-question survey that included current diagnostic and therapeutic approaches for renal colic during pregnancy and known controversies. Renal colic in this study was considered to be caused by urolithiasis and did not include physiological hydronephrosis. The questionnaire was titled "Investigation of Chinese urologists’ preferences for diagnosing and treating renal colic during pregnancy", and it consisted of two (yes/no) to seven options. For questions 10, 11, 13, and 17, respondents were allowed to check 1–3 boxes. For the remaining questions, the respondents were allowed to check only 1 box. All the questions had to be answered before the questionnaire was finally submitted.

The questions inquired about the following information: the background and professional experience or knowledge of potential respondents; the number of patients admitted with renal colic during pregnancy; the method of diagnosing and treating renal colic during pregnancy; and possible complications of surgical intervention for renal colic during pregnancy.

The urologists completed the questionnaire, and the results were anonymous to accurately understand the current status of diagnosing and treating gestational renal colic. WeChat, China's largest social platform, was used to distribute the questionnaire among urologists at hospitals of all levels in China. This study was conducted from April 30, 2022, to May 30, 2022. Given that this voluntary survey involved colleagues but not patients, the requirement for ethics approval was waived by the Ethics Committee of the First Affiliated Hospital of University of South China. However, informed consent was still obtained from all the participants by filling the survey. All methods were performed in accordance with the relevant guidelines and regulations. The original survey questionnaire used for the present study was written in Chinese, and the translated English version of the questionnaire is provided in the Supplementary material.

The Statistical Package for Social Science software (version 25.0; IBM, USA) was used for descriptive statistics and survey analysis. To investigate the associations between urologists with different professional titles and the variables (1) use of diagnostic imaging tools, (2) medication selection for conservative treatment, and (3) choice of surgical methods, chi-square and Fisher’s exact tests of associations were used. All significant (*p* < 0.05) associations were subsequently investigated using the Bonferroni multiple comparisons test to determine directionality.

## Results

Three hundred Chinese urologists from hospitals of all levels were contacted by the study team, and 110 of them responded. Most of the respondents were between 30 and 39 years old. The majority (68.2%) of respondents worked in Grade A tertiary hospitals, and doctors with doctoral or master’s degrees (20.0% and 48.2%, respectively) accounted for the majority of respondents. There were 31 (28.2%), 30 (27.3%), 36 (32.7%), and 13 (11.8%) respondents with less than 5 years, 6–10 years, 11–20 years, and more than 20 years of experience, respectively. Of the 110 respondents, 30 (27.3%), 44 (40.0%), 30 (27.3%), and 6 (5.5%) were resident urologists, attending urologists, deputy chief urologists, and chief urologists, respectively (Table 1).

**Table 1:** Respondent demographics.

Characteristics	No. of urologists	Percentage, %
Age(year)		
< 30	20	18.2
30–39	57	51.8
40–49	25	22.7
≧50	8	7.3
Hospital setting		
Grade A tertiary hospital	75	68.2
Grade B tertiary hospital	8	7.3
Secondary hospital	25	22.7
Inferior to secondary hospital	2	1.8
Professional title		
Resident doctors	30	27.3
Attending doctors	44	40.0
Associate chief doctors	30	27.3
Chief doctors	6	5.5
Degree		
M.D	22	20
Master	53	48.2
Undergraduate	32	29.1
Inferior to undergraduate	3	2.7
Work experience (year)		
< 5	31	28.2
6–10	30	27.3
11–20	36	32.7
> 20	13	11.8

Among the 110 urologists who responded, 64 (58.2%) admitted less than 10 pregnant women with renal colic each year, 33 (30.0%) admitted 10–20 patients per year, and 7 (6.4%) admitted 20–30 patients per year. Only 6 (5.5%) respondents reported admitting more than 30 pregnant women with renal colic per year.

**Figure 1:**
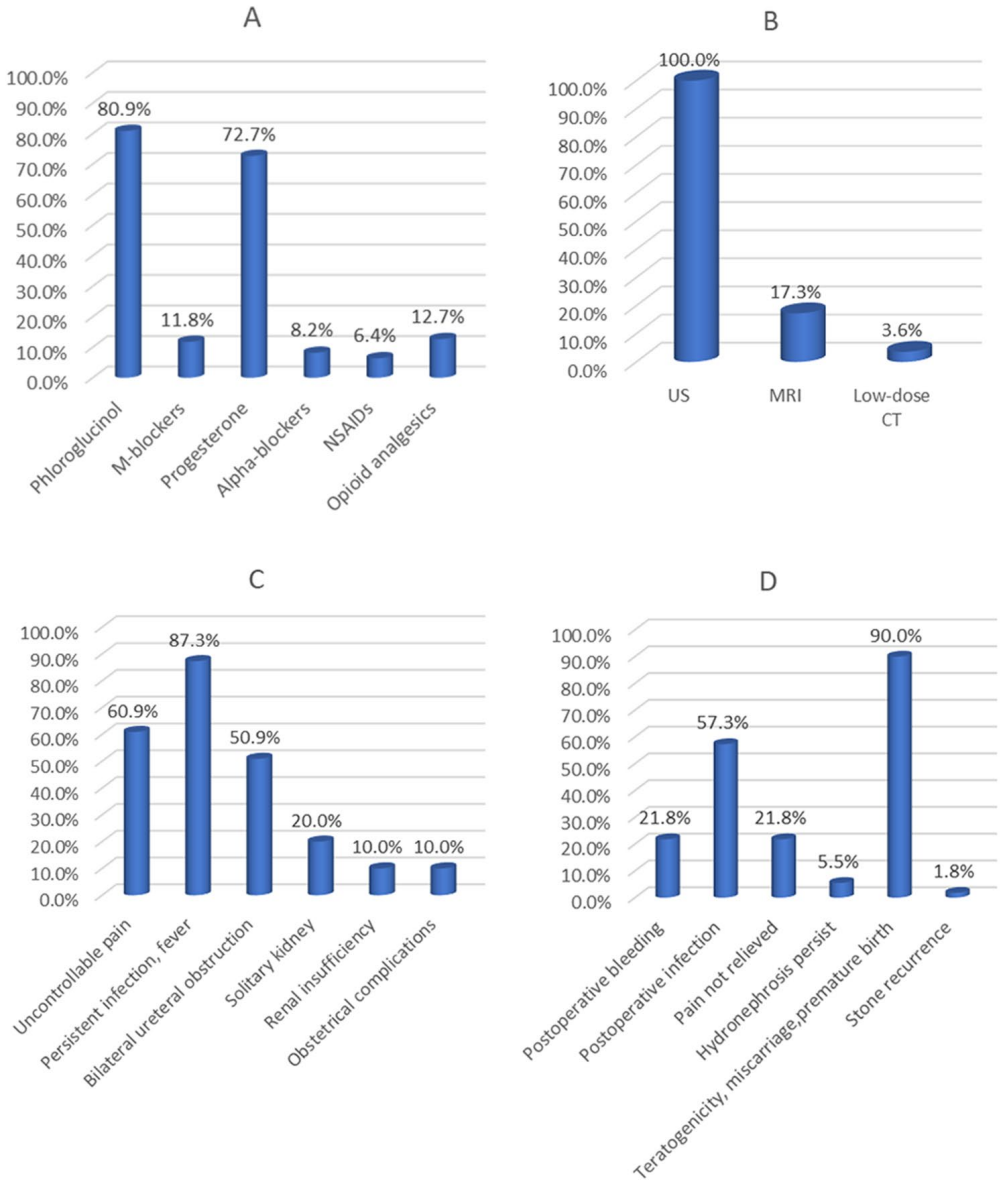
(**A**) Antispasmodic and analgesic use. (**B**) Diagnostic tool use. (**C**) Indications for surgery. (**D**) Concerns of urologists.

**Figure 2:**
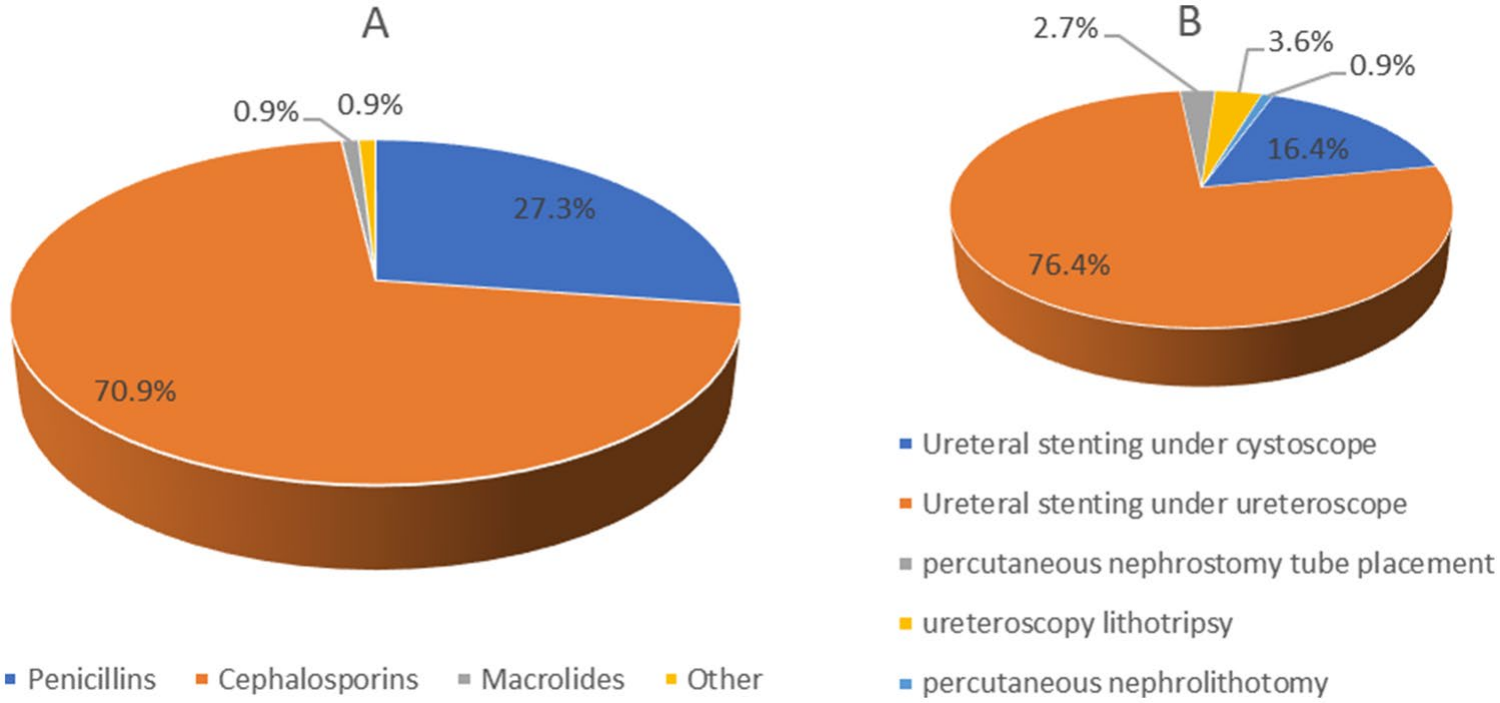
(**A**) Antibiotic use. (**B**) Use of surgical procedures.

All (100.0%) respondents used US, 19 (17.3%) respondents used MRI, and 4 (3.6%) respondents used low-dose CT (Fig. 1B). In addition, all (100.0%) respondents indicated that patients with renal colic during pregnancy were initially treated conservatively. Regarding anti-inflammatory treatment (in case of infection), 70.9% of the respondents used cephalosporins, 27.3% of the respondents used penicillin, and only 0.9% of the respondents used macrolides (Fig. 2A). Of all the respondents, 89 (80.9%) used smooth muscle antispasmodics (phloroglucinol) for pain relief, 13 (11.8%) used antimuscarinics (M receptor blockers) for pain relief, 80 (72.7%) used progesterone for pain relief, 7 (6.4%) used nonsteroidal anti-inflammatory drugs (NSAIDs) for pain relief, and 14 (12.7%) used opioid analgesics for pain relief. In addition, 9 (8.2%) respondents indicated that they would use alpha-adrenoreceptor blockers (Fig. 1A).

In total, 70 respondents (63.6%) indicated that conservative treatment was ineffective in 0–20% of patients, 24 respondents (21.8%) indicated that 20–40% of patients required surgical treatment, and only 16 respondents (14.5%) indicated that more than 40% of patients required surgical treatment. In addition, most urologists (87.3%) indicated that the reason for surgical intervention was persistent infection or fever followed by uncontrollable pain (60.9%), bilateral ureteral obstruction (50.9%), solitary kidney (20%), or renal failure or obstetric complications (only 10%) (Fig. 1C). For the choice of procedure, most of the urologists (76.4%) preferred to place a ureteral stent under the guidance of a ureteroscope, followed by placement under the guidance of a cystoscope (16.4%). Percutaneous nephrostomy tube placement, ureteroscopy lithotripsy, and percutaneous nephrolithotomy were the preferred methods used by 2.7%, 3.6%, and 0.9% of the urologists, respectively (Fig. 2B). In addition, 96.4% of the urologists believed that routine ureteral stent placement after surgery was necessary. The main factors influencing the choice of surgical method were the patient's gestational week (53/110, 48.2%), surgical risk (47/110, 42.7%) and postoperative complications risks (8/110, 7.3%). Most urologists were concerned about postoperative complications, such as malformation, miscarriage, or preterm delivery (99/110, 90.0%), as well as postoperative infection (63/110, 57.3%) and postoperative bleeding (24/110, 21.8%) (Fig. 1D).

Moreover, there were no differences in the choices of urologists with different professional titles regarding diagnostic tools, most therapeutic medications, or surgical methods (*p* > 0.05) (Table 2). Interestingly, a significant difference was found in the use of alpha-adrenoreceptor blockers between doctors with different professional titles (*p* = 0.029), with chief urologists using alpha-adrenoreceptor blockers more frequently than resident urologists did.

**Table 2:** Effect of professional title of urologist against preference for diagnosing and treating renal colic combined with pregnancy.

Variable	Total (n = 110)	Professional title				*p*-Chi^2^	*p*-value
		Resident doctors (n = 30)	Attending doctors (n = 44)	Associate chief doctors (n = 30)	Chief doctors (n = 6)		
Diagnostic imaging tools							
US	110	30	44	30	6	–	–
MRI	19	5	5	9	0	4.80	0.154^a^
Low-dose CT	4	3	1	0	0	3.81	0.299^a^
Antibiotics							
Penicillins	30	10	12	7	1	1.03	0.810^a^
Cephalosporins	78	18	32	23	5	2.50	0.489^a^
Macrolides	1	1	0	0	0	3.54	0.600^a^
Others	1	1	0	0	0	3.54	0.600^a^
Analgesia							
Phloroglucinol	89	20	37	27	5	5.43	0.116^a^
M receptor blockers	13	7	4	2	0	4.39	0.189^a^
Progesterone	80	20	33	25	2	6.54	0.073^a^
Alpha-adrenoreceptor blockers	9	0	3	4	2	8.07	0.029^a^
NSAIDs	7	0	5	2	0	3.69	0.268^a^
Opioid analgesics	14	4	6	4	0	0.49	1.000^a^
Others	1	1	0	0	0	3.54	0.600^a^
Surgical intervention							
Renal drainage	105	28	42	29	6	0.81	1.000^a^
Definitive stone treatment	5	2	2	1	0	0.81	1.000^a^
Renal drainage							
Ureteral stenting under the guidance of a ureteroscope	84	22	33	23	6	1.68	0.684^a^
Ureteral stenting under the guidance of a cystoscope	18	5	9	4	0	1.33	0.766^a^
Percutaneous nephrostomy	3	1	0	2	0	3.31	0.315^a^
Definitive stone treatment							
Ureteroscopy lithotripsy	4	2	1	1	0	1.48	0.853^a^
Percutaneous nephrolithotomy	1	0	1	0	0	2.77	1.000^a^

US, Ultrasonography; MRI, Magnetic resonance imaging; CT, Computed tomography; NSAIDs, Nonsteroidal anti-inflammatory drugs;

^a^Fisher’s exact test.

## Discussion

The methods for diagnosing and treating renal colic during pregnancy vary, and they are the subjects of debate, as the selected modes depend on personal preference and comprehensive assessment by the doctor. To the best of our knowledge, this is the first survey to focus on urologists’ preferences in selecting modes for diagnosing and treating pregnant women with renal colic.

In this study, most of the respondents worked in Grade A tertiary hospitals. More than half of the respondents had master's or doctoral degrees, and nearly half of the respondents had more than 10 years of clinical experience. Most urologists reported admitting less than 10 pregnant women with renal colic per year, suggesting that the incidence of renal colic during pregnancy is not high, which aligns with the findings of a previous study^2^.

The results of the questionnaire indicated that all doctors preferred to use US. US is noninvasive, requires no radiation, and does not affect foetal development, making it the primary radiological diagnostic tool. These results were in line with the recommendations of the AUA and EAU guidelines^11,12^. However, a previous study has shown that physiologic hydronephrosis during pregnancy may hinder diagnosis, and the sensitivity and specificity of US are both reduced and insufficient for accurate diagnosis^13^. Therefore, US alone may not be sufficient, and other auxiliary tools should be combined if necessary. MRI should be considered a second-line diagnostic tool^14^. According to the results of the questionnaire, 19 urologists (17.3%) used MRI. A previous study showed that US combined with low-dose CT has a higher positive rate than US combined with MRI^15^. Although studies have shown that low-dose CT confers a low risk of foetal harm and is a safe and high-precision imaging technique^7,16^, the use of low-dose CT is still debated. Only 4 respondents (3.6%) selected low-dose CT in this study, which showed that low-dose CT has not been widely accepted by Chinese urologists as a method of diagnosing renal colic during pregnancy.

Once diagnosed with ureteral calculi, most patients receive conservative treatment^17^, including antispasmodics and pain relievers as well as anti-infection (in case of infection) and symptomatic treatment. The present study revealed that the most frequently used drug was phloroglucinol (80.9%) followed by progesterone (72.7%). Phloroglucinol is a highly selective drug that is released in abnormal spastic smooth muscle, and effectively relieves abnormal uterine contraction and ureteral spasm but does not cause malformation of the foetus. Phloroglucinol is widely used for acute abdomen during pregnancy^18^. Progesterone relaxes ureteral smooth muscle and inhibits uterine contractions, and it is often used as the drug of choice for treating urinary calculi during pregnancy. However, progesterone has potential cardiovascular effects and should be used with caution in pregnant patients complicated with hypertension. In addition, fewer urologists selected M receptor blockers to relieve spasm (11.28%). A previous study reported that opioids are the main analgesics used for conservative treatment^2^. Notably, the present survey showed that only 14 urologists (12.7%) opted for opioid analgesics, which may potentially affect foetal development and teratogenicity^19^. In the present study, 9 urologists (8.2%) used alpha-adrenoreceptor blockers, which are common drugs used in medical expulsive therapy (MET). Compared to resident doctors, chief doctors seem to prefer the use of alpha-adrenergic blockers. However, the safety of MET in the treatment of urinary calculi during pregnancy still lacks sufficient research support. A small number of respondents (6.4%) selected NSAIDs, which should be used with caution because they may lead to premature closure of the foetal ductus arteriosus, premature delivery, or abortion^20^. A multidisciplinary consultation should be considered before prescribing NSAIDs to avoid such risks. Where antibiotics are indicated, penicillin and cephalosporins may be the safest choices^21^. However, the present study showed that cephalosporins were used more frequently (70.9%) than penicillin was (27.3%). Aminoglycosides, tetracycline, chloramphenicol, fluoroquinolones, and sulfa antibiotics are contraindicated during pregnancy^5^.

Although conservative management is the first consideration, 26–30% of pregnant women still have to undergo surgery for conditions such as uncontrollable pain, severe infection, bilateral ureteral obstruction, obstetrical complications, renal insufficiency, or a solitary kidney with obstruction^2,8^. The present survey revealed that persistent infection or fever was the most common condition (87.3%) followed by uncontrollable pain (60.9%), and obstetric complications were uncommon (11.10%). In addition, the majority of physicians (63.6%) indicated that no more than 20% of the patients they treated required surgery.

If surgical intervention is necessary, renal drainage or definitive stone treatment should be chosen according to the patient's condition to increase safety and achieve the most ideal therapeutic results. However, the choice of specific treatment has always been debated^22^. Ureteral stenting and percutaneous nephrostomy tube placement can release the obstruction and drain urine smoothly, and both options are recommended in the AUA and EAU guidelines due to minimal trauma and high efficiency^11,12^. However, the present survey revealed that most urologists prefer ureteral stenting (92.7%) to percutaneous nephrostomy (2.7%), which aligned with the findings of a previous study^23^. Percutaneous nephrostomy is an invasive operation with some disadvantages, as follows: discomfort caused by the fistula in the lumbar region; inability to lie flat on the stoma side; the presence of external drains makes care difficult; and susceptibility to bacterial colonization, infection, lumen obstruction, erosion, and bleeding^24,25^. Ureteral stenting is considered to be the least invasive surgical procedure and is given priority. However, ureteral stent implantation may fail^16^. If this occurs, percutaneous nephrostomy should then be considered. In addition, both methods may require drainage tube replacement every 4–6 weeks during the remainder pregnancy due to high rates of encrustation in pregnant women resulting from metabolic changes^26^.

With the progress of endoscopic equipment, lithotripsy technology, and anaesthesia, temporary renal drainage has been replaced by definite stone treatment in some centres^1,27^. The present survey revealed that 4.5% of urologists preferred definite stone treatment, including ureteroscopy lithotripsy (3.6%) and percutaneous nephrolithotomy (0.9%). Definite stone treatment is usually indicated for the following patients: patients with long-term indwelling nephrostomy tubes or ureteral stents; patients who are unable to tolerate or unwilling to undergo nephrostomy tube and ureteral stent implantation; and patients with multiple calculi in the kidney. Ureteroscopy is safe for the treatment of urolithiasis during pregnancy^28^. However, percutaneous nephrolithotomy is the subject of debate and is not recommended during pregnancy^29^. In addition, uterine compression and elevated progesterone levels during pregnancy cause physiological dilation of the ureter above the pelvic rim, facilitating ureteroscopy lithotripsy without dilation^30^. In addition, most urologists are concerned about postoperative complications, such as malformation, miscarriage/premature delivery (90.0%), postoperative infection (57.3%), and postoperative bleeding (21.8%).

In the Chinese health care system, urologists are categorized into three levels based on their practice and experience: junior (resident urologists), intermediate (attending urologists), and senior (associate chief and chief urologists). Generally, it is believed that a higher professional title is correlated with more extensive experience. Given the importance and complexity of renal colic during pregnancy, this study investigated the potential variances in diagnostic and therapeutic preferences among urologists with different professional titles. The analysis revealed that while chief urologists seem to prefer the use of alpha-adrenergic blockers in conservative management more frequently than resident urologists did, there was overarching consistency in the diagnostic and treatment preferences for renal colic during pregnancy among urologists of different professional titles.

This study has several limitations. First, the response rate was relatively low, leading to a relatively small sample size. Second, the results of the present survey could not guarantee that the answers selected by urologists were always consistent with actual clinical practice as many factors in clinical practice may affect final decisions. Third, the present survey could not guarantee that the respondents who participated in this survey were representative of the general population of urologists in China based on various factors, such as professional title and hospital setting.

## Conclusion

The present study improved our understanding of the diagnosis and treatment strategies for patients with renal colic during pregnancy in China. US is the preferred diagnostic tool, followed by MRI. However, low-dose CT is not widely accepted. Patients with renal colic during pregnancy are initially treated conservatively. Phloroglucinol and progesterone are the most commonly used antispasmodics and analgesics. Cephalosporins are the most commonly used antibiotics, whereas opioid analgesics are not commonly used. If surgery is unavoidable, most urologists prefer temporary renal drainage, employing ureteral stenting prior to percutaneous nephrostomy. Overall, Chinese urologists with different professional titles have similar preferences for diagnosing and treating renal colic during pregnancy.

### Supplementary Information

Supplementary Information.

## Data Availability

The datasets generated during and/or analysed during the current study are available from the corresponding author on reasonable request.

## Abbreviations

US: Ultrasonography
MRI: Magnetic resonance imaging
CT: Computed tomography
MET: Medical expulsive therapy
NSAIDs: Nonsteroidal anti-inflammatory drugs
