# Supplementary material for: Preference for diagnosing and treating renal colic during pregnancy: a survey among Chinese urologists
Source: Sci Rep. 2024 Feb 5;14:2914. doi: 10.1038/s41598-024-53608-w (PMC10844619; doi:10.1038/s41598-024-53608-w)
Supplement: Supplementary file 1 — Supplementary Information. [file 41598_2024_53608_MOESM1_ESM.docx]

**Investigation of Chinese urologists’ preferences for diagnosing and treating renal colic during pregnancy**

**1. Your age. (year)**

A.<30

B.30–39

C.40–49

D.50–59

E.≧60

**2. Hospital setting where you work.**

A. Grade A tertiary hospital

B. Grade B tertiary hospital

C. Secondary hospital

D. Inferior to secondary hospital

**3. What’s your practice role?**

A. Resident doctors

B. Attending doctors

C. Associate chief doctors

D. Chief doctors

**4. What is your academic background?**

A. M.D.

B. Master

C. Undergraduate

D. Inferior to undergraduate

**5. Are you a urologist?**

A. Yes

B. No

**6. How long have you been working?**

A. < 5 years

B. 6-10 years

C. 11-20 years

D. > 20 years

**7. Approximately how many pregnant women with renal colic do you (or your department) admit each year?**

A. < 10

B. 10-20

C. 20-30

D. > 30

**8. What is the first treatment you would consider for renal colic in pregnancy?**

A. Conservation management

B. Surgical intervention

**9. Which antibiotic do you routinely prefer for pregnant women with renal colic? (If needed)**

A. Penicillins

B. Cephalosporins

C. Macrolides

D. Others

**10.** **Which antispasmodic and pain medications do you use most often for pregnant women with renal colic? (Choose 1-3 items)**

A. Smooth muscle antispasmodics (phloroglucinol, etc.)

B. M receptor blockers

C. Progesterone

D. Alpha-adrenoreceptor blockers

E. NSAIDs

F. Opioid analgesics

G. Others

**11. What radiological diagnostic tools will you use** **for pregnant women with renal colic? (Choose 1-3 items)**

A. Ultrasonography

B. Magnetic resonance imaging

C. Low-dose CT

D. Others

**12. What percentage of pregnant women with renal colic do you admit that require surgical treatment?**

A. 0-20%

B. 20-40%

C. 40-60%

D. 60-80%

E. 80-100%

**13. What are the most important reasons for surgical treatment of pregnant women with renal colic that you admit? (Choose 1-3 items)**

A. Uncontrollable pain

B. Persistent infection or fever

C. Bilateral ureteral obstruction

D. Solitary kidney

F. Renal failure

G. Obstetric complications

**14. Which surgical treatment option do you usually take for pregnant women with renal colic that you admit?**

A. Place JJ stent under the guidance of a ureteroscope

B. Place JJ stent under the guidance of a cystoscope

C. Percutaneous nephrostomy tube placement

D. Ureteroscopy lithotripsy

F. Percutaneous nephrolithotomy

**15. What is the main factor that influenced your surgical plan decision?**

A. Surgical risk

B. Postoperative complications

C. Operator experience

D. Patient's gestational age

E. Others

**16.** **Do you routinely leave a JJ stent in place after surgery?**

A. Yes

B. No

**17. Which of the following conditions are you most concerned about after surgery? (Choose 1-3 items)**

A. Postoperative bleeding

B. Postoperative infection

C. The pain can’t be relieved.

D. Hydronephrosis can’t be alleviated.

E. Teratogenicity, miscarriage, preterm delivery

F. Stone recurrence

G. Others
